# Assessing trade-offs to inform ecosystem-based fisheries management of forage fish

**DOI:** 10.1038/srep07110

**Published:** 2014-11-19

**Authors:** Andrew Olaf Shelton, Jameal F. Samhouri, Adrian C. Stier, Philip S. Levin

**Affiliations:** 1 Conservation Biology Division, Northwest Fisheries Science Center, National Marine Fisheries Service, National Oceanic & Atmospheric Administration, Seattle, WA

## Abstract

Twenty-first century conservation is centered on negotiating trade-offs between the diverse needs of people and the needs of the other species constituting coupled human-natural ecosystems. Marine forage fishes, such as sardines, anchovies, and herring, are a nexus for such trade-offs because they are both central nodes in marine food webs and targeted by fisheries. An important example is Pacific herring, *Clupea pallisii* in the Northeast Pacific. Herring populations are subject to two distinct fisheries: one that harvests adults and one that harvests spawned eggs. We develop stochastic, age-structured models to assess the interaction between fisheries, herring populations, and the persistence of predators reliant on herring populations. We show that egg- and adult-fishing have asymmetric effects on herring population dynamics - herring stocks can withstand higher levels of egg harvest before becoming depleted. Second, ecosystem thresholds proposed to ensure the persistence of herring predators do not necessarily pose more stringent constraints on fisheries than conventional, fishery driven harvest guidelines. Our approach provides a general template to evaluate ecosystem trade-offs between stage-specific harvest practices in relation to environmental variability, the risk of fishery closures, and the risk of exceeding ecosystem thresholds intended to ensure conservation goals are met.

Harvesting natural populations has both intended and unintended consequences. The intended consequences result from the extraction of biomass from an ecosystem for use by people; timber, wild seafood, and game serve as familiar examples. The unintended consequences of harvest, however, are highly variable and can include evolutionary shifts in populations, habitat modification, incidental mortality of non-target species, altered food web interactions, and changes in ecosystem functions and services^1^,^2^. This potential for far-reaching problems underlies emerging enthusiasm for ecosystem-based management, which is predicated on addressing connections between people's activities, species, and ecosystems^3^,^4^. It also overlooks the more immediate, and often unresolved, danger of managing conflicts that can arise due to the cumulative impacts of multiple human influences on a single species^5^. If activities cannot be successfully coordinated within any single use sector, it would seem exceedingly ambitious to expect that activities might be coordinated across sectors^6^.

Individual species prosecuted by multiple fisheries provide an interesting case in point. There are many stocks worldwide that are exploited by more than one fishing gear, in more than one season, and/or during more than one life stage. From an ecological perspective, harvest of stage-structured populations increases the complexity of dynamics^7^. Dozens of fishes are prized both for their roe (eggs) and for the protein derived from adult life stages (e.g. salmon (*Oncorhynchus spp.*); herring (*Clupea spp.*), sturgeon (*Acipenseridae*), shad (*Alosa spp.*)). Several aquatic reptiles and birds are exploited both for their eggs and for the meat derived from adult life stages (e.g., turtles, alligators, gulls). The consequences of such stage-specific exploitation may lead to non-equivalent effects of alternate management strategies on the target species (e.g.^8^).

Harvesting young, pre-reproductive individuals will generally result in qualitatively different population dynamics than harvesting mature adults^9^,^10^. This non-equivalency can produce trade-offs that influence both the ecosystem service of (sea)food provisioning and the socio-economic status of fishers and fishing communities. Surprisingly, there are many stocks lacking coordination of harvest strategies among fisheries affecting different life stages^11^,^12^, perhaps because the fisheries occur in different geographies, at different times of year, or both.

This lack of coordination is one factor underlying calls to move toward ecosystem-based fisheries management (EBFM^2^;). Adding to this rationale, the potential for trade-offs among fisheries targeting the same stock at different life stages is complicated by a third axis of conservation importance: the persistence of the exploited species. Recent publications have focused on conservation motivated by the intrinsic value of species, and the moral imperative to avoid extinctions^13^,^14^,^15^,^16^. Thus removal of fish biomass by fisheries can threaten this conservation aim if it proceeds too close to full depletion of any individual stock. In addition, and as importantly, policy directives around the world (e.g.^17^,^18^,) now include conservation-oriented goals for EBFM that acknowledge incidental effects of fishing on the predators and prey of exploited stocks.

Marine forage fish provide a focal point for these issues because of the ecosystem implications of their harvest^19^. Forage fish are small to intermediate-sized pelagic species (e.g. sardine, anchovy, sprat, herring, capelin, krill, squid) that serve as the chief link between the lower trophic levels on which they feed (zooplankton and phytoplankton) and the upper trophic level predators (mammals, birds, larger fishes) that rely upon them as a primary food source^20^. They also tend to be characterized by dramatically fluctuating populations^21^,^22^ with the preponderance of the evidence indicating that stochastic environmental forcing plays a strong role^23^,^24^,^25^. It has been argued that the value of forage fish to other fisheries can be quite large (e.g.^26^), including a recent global estimate that they are worth twice as much as fisheries directed at forage fish themselves^20^. Furthermore, recent attempts to quantify the importance of forage fish to non-exploited species such as seabirds suggest that forage fish biomass should be maintained at levels higher than typically set as part of fisheries harvest guidelines^22^,^27^, though such ecosystem thresholds for forage fisheries have not yet been widely adopted. Less effort has been devoted to evaluating how such ecosystem constraints may trade-off with fishery priorities at a spatial scale relevant to forage fish management.

Pacific herring (*Clupea pallasii*), a forage fish species occurring in the North Pacific, are emblematic of the need to understand the population dynamic consequences of alternative forms of exploitation in order to achieve sustainable fisheries and conservation goals. Off the coast of British Columbia and Alaska, Pacific herring are subject to at least two distinct fisheries: one that catches mature spawning adults and one that harvests (immature) eggs. Ecological principles suggest that the two fisheries have distinct influences on herring dynamics, but the interaction between multiple fisheries and ecosystem considerations is largely unexplored. In this paper, we develop a stochastic, age-structured model to investigate the consequences of egg- and adult-harvest for population dynamics, fisheries, and conservation. In particular, we focus attention on how different combinations of harvest intensity affect herring biomass (in relation to both species-specific and ecosystem thresholds), the catch of adult herring and eggs, and the frequency of fishery closures. Our approach provides a template to evaluate trade-offs between egg- and adult-harvest rates in relation to environmental variability, risk to fisheries in terms of the probability of fishery closures, and the risk to ecosystems based on the dietary needs of predators reliant upon exploited stocks.

## Results

**Figure 1:**
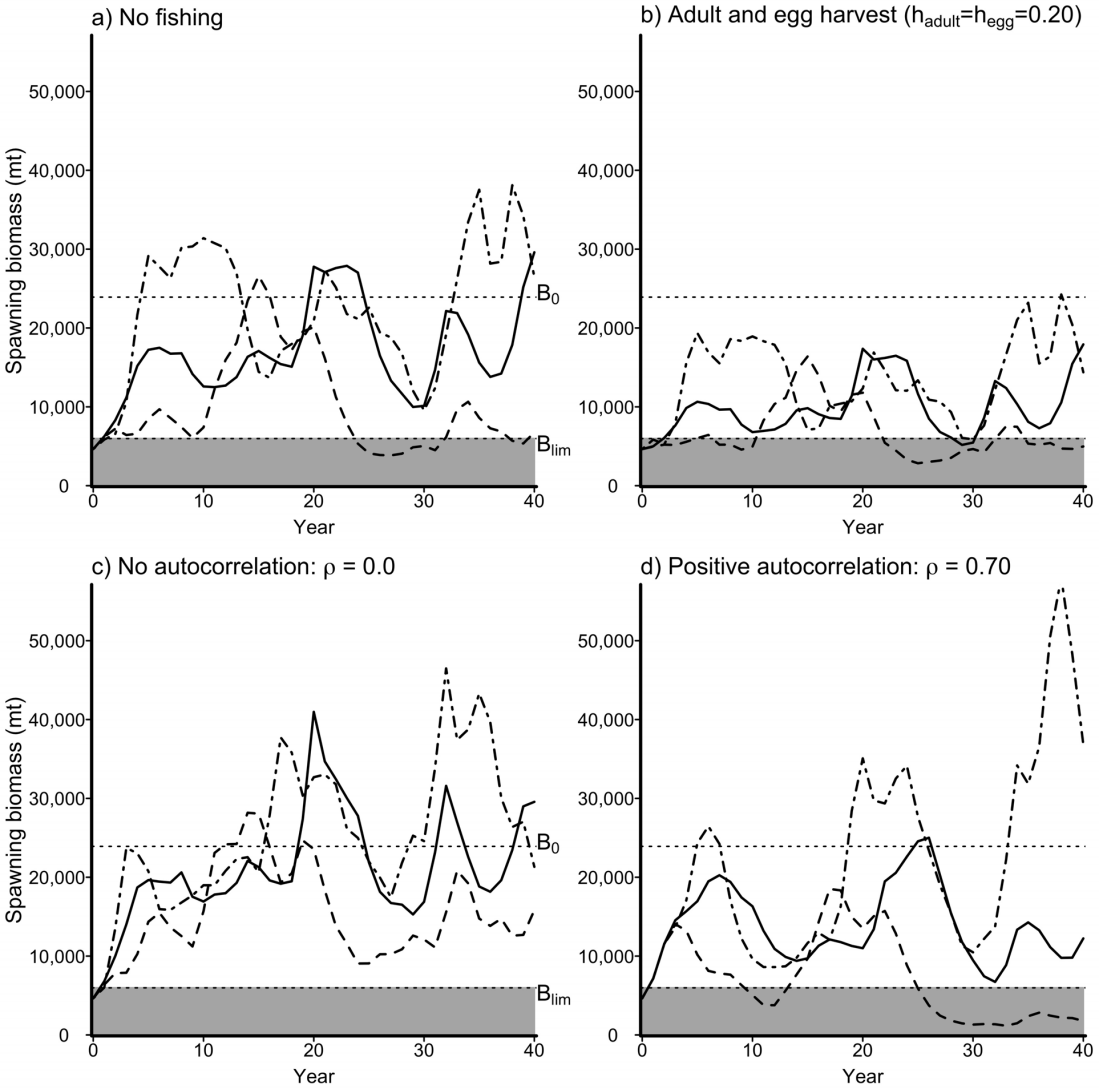
Examples of simulated herring time-series. Three simulated time series from the age-structured model: (*a*) simulations with no fishing (*b*) simulations with moderate levels of herring harvest. Both (*a*) and (*b*) were conducted with *CV* = 0.8 and *ρ* = 0.5. Panels (*c*) and (*d*) show simulation in the absence of fishing but contrasting levels of temporal autocorrelation in recruitment: (*c*) simulations with no temporal autocorrelation in recruitment *ρ* = 0.0, (*d*) three simulations with strong positive autocorrelation *ρ* = 0.7. Both (*c*) and (*d*) were conducted with *CV* = 0.8. In all panels, grey shaded area indicates biomass levels at which the fishery is closed.

Herring population dynamics are strongly influenced by fishing (Fig. 1a, 1b) and recruitment regimes (Fig. 1c, 1d). We present results for simulations that assume a moderate level of recruitment variability (coefficient of variation, *CV* = 0.8, lag-one autocorrelation, *ρ* = 0.5); results under alternate simulation recruitment scenarios are provided in the Supplementary materials.

**Figure 2:**
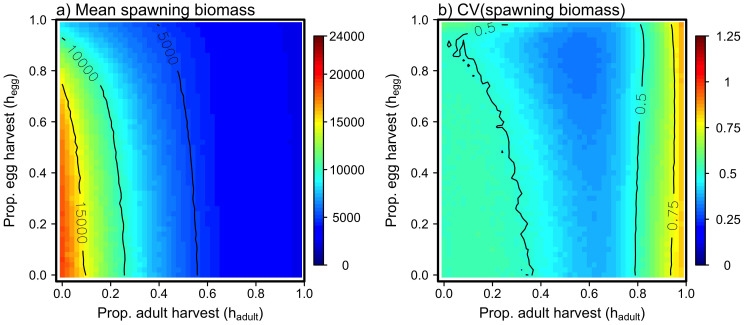
Consequences of adult and egg harvest strategies on mean spawning biomass (*a*) and the coefficient of variation in spawning biomass (*b*). Both panels show results for CV = 0.8, and *ρ* = 0.5.

Annual harvest of both adults and eggs each can strongly affect herring spawning biomass (Fig. 2). Increasing either adult or egg harvest results in decreased mean biomass during the 40 year span of the simulation; however, populations show distinct responses to harvest on eggs relative to harvest on adults. Increasing harvest on adults, *h_adult_*, causes a rapid decline in mean spawning biomass – high harvest rates on adults (*h_adult_* >≈ 0.50) can result in mean biomass below the fishery closure limit *B_lim_* (~5,900 mt, Table 1). In contrast, harvest on eggs, *h_egg_*, by itself has a more limited effect on mean biomass until the annual proportion harvested, *h_egg_*, > 0.70; indeed, until *h_egg_* > 0.90 mean biomass always exceeds 10,000 mt. The cooefficient of variation of biomass, *CV_SSB_*, is relatively stable across harvesting scenarios (generally 0.5 < *CV_SSB_* < 0.75). The only exception is an area in which *CV* < 0.5 that corresponds to high adult harvest rates or high egg harvest and moderate to high adult harvest in which the population fluctuates very near *B_lim_* (Fig. 2b).

**Figure 3:**
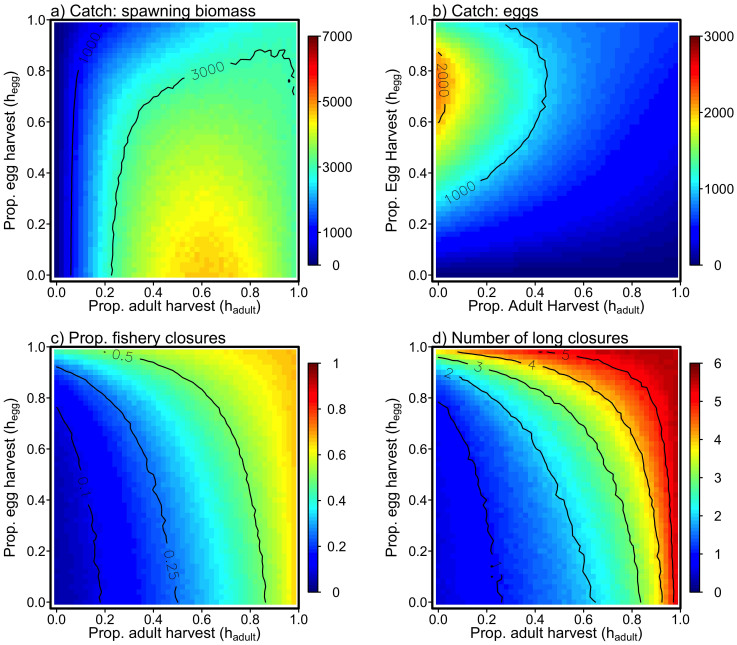
Consequences of adult and egg harvest strategies on the herring fisheries. (*a*) Mean catch of spawning biomass (mt), (*b*) mean catch of eggs (trillions), (*c*) the proportion of years that the fishery is closed, and (*d*) mean number of long closures (>3 consecutive years closed) in the 40 year simulation.

There is a strong and asymmetric trade-off between the catch of adults and catch of eggs (Fig. 3a, 3b). Overall, high egg harvest rates do not allow for high adult catch and vice versa. Mean catch of eggs is maximized at a very high proportional harvest rate (*h_egg_* ≈ 0.7) while adult harvest rate is maximized at *h_adult_* ≈ 0.60. However the interaction between adult and egg harvest is notably asymmetric; for any level of egg harvest, slightly increasing *h_adult_* results in a dramatic decline in mean egg catch. In contrast, the effect of egg harvest on adult catch is relatively minor. Only large changes in *h_egg_* can noticeably affect the mean catch of adult fish.

**Figure 4:**
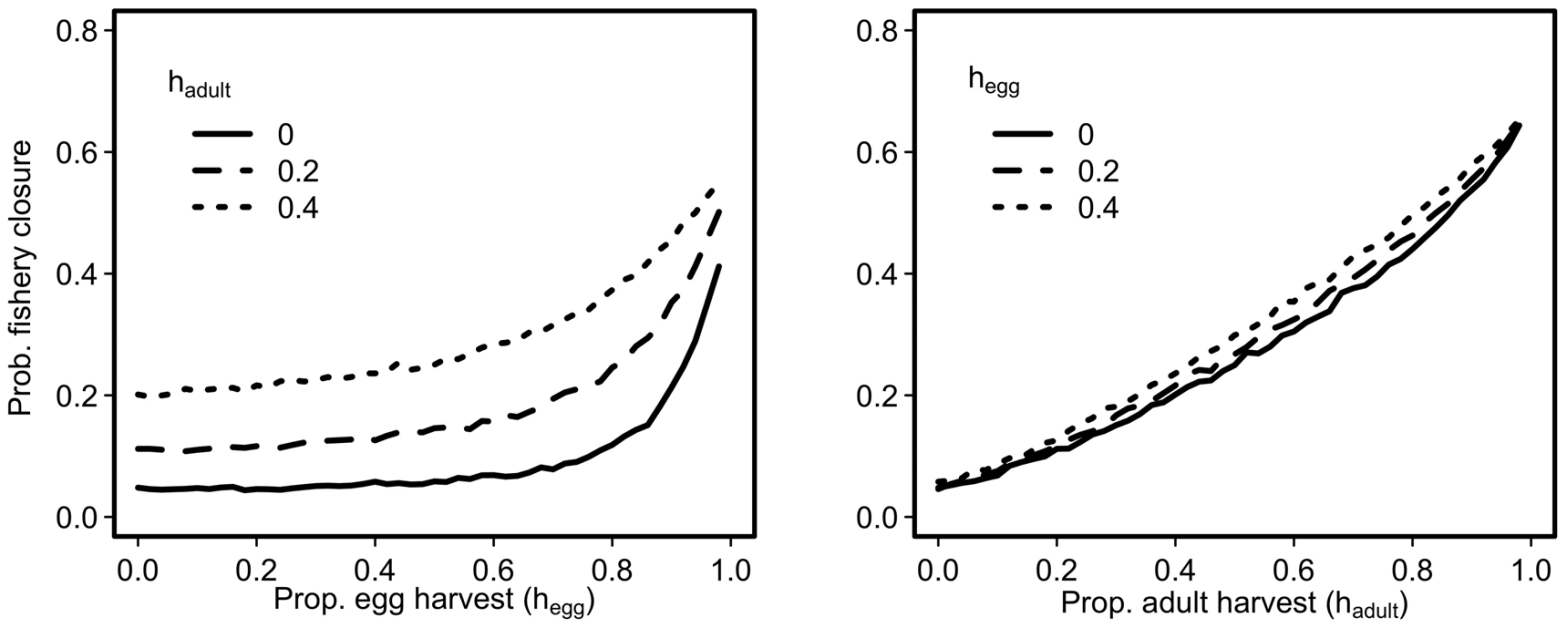
The consequences for the probability fishery closure of changing egg harvest for three fixed levels of adult harvest (left panel) and changing adult harvest for three fixed levels of egg harvest (right panel).

Large mean catches arising from high harvest rates come with a cost: greater variation in catch. With harvest rates that yield high average catches, the spawning biomass is frequently pushed below *B_lim_* and the fishery closes (Fig. 3c). Closures occur because the fish population has been reduced to a low level (below 0.25*B_0_* in this case), causing a control rule to kick in such that no adults or eggs can be harvested. For example, in all scenarios with *h_adult_* ≈ 0.65, the fishery is closed more than 25% of the time (Fig. 3c). Furthermore, these fishery closures tend to occur for extended periods (at least 1 fishery closure of at least 3 years in duration closed is expected; Fig. 3d). Increasing frequency of closures is more strongly affected by harvest of adults (Fig. 4a) than the harvest of eggs (Fig. 4b). However, fishery closure rates of greater than 10% are associated with *h_egg_* that maximizes mean catch (Figs. 3b,c).

**Figure 5:**
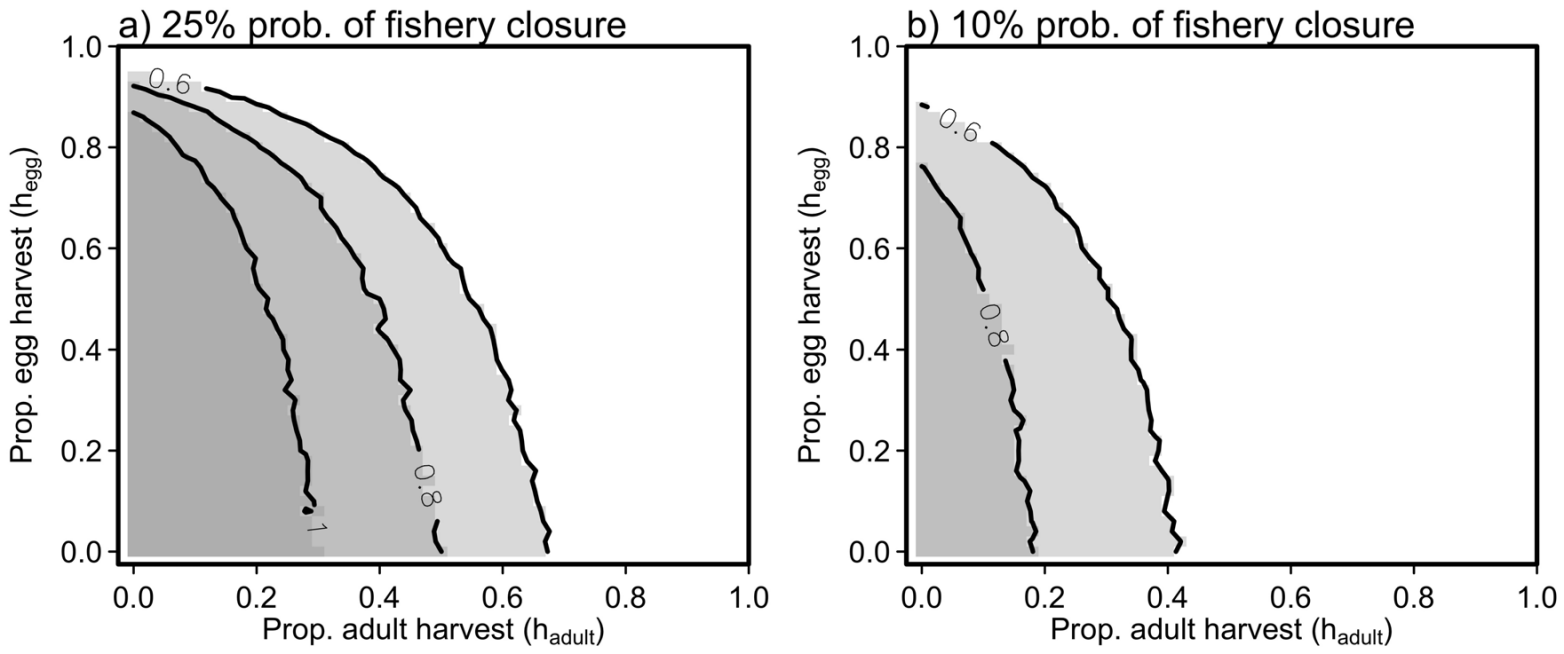
Risk plots for two probability of fisheries closure under harvesting scenarios. Shaded areas and isoclines indicate levels of harvest that maintain below a specified risk tolerance for three different levels of recruitment variability (*CV* = 0.6, 0.8 or 1.0) and *ρ* = 0.5. (*a*) Isoclines for 25% probability of fishery closure. (*b*) Isoclines for 10% probability of fishery closure. Note that that there are no scenarios with *CV* = 1.0 that result in <10% probability of fishery closure in panel (*b*).

An important aspect of assessing trade-offs between the egg and adult herring fisheries is to document how risk of fishery closure can change in the face of recruitment uncertainty. Harvesting strategies that achieve a fishery closure rate of less than 25% of years (Fig. 5a) and less than 10% (Fig. 5b) change substantially under different recruitment scenarios. In particular, increasing recruitment *CV* means fewer combinations of harvest rates meet fishery closure targets. Stated another way, under identical fisheries pressure, populations with high recruitment variability will decline below *B_lim_* more frequently than populations with small recruitment variability. For the simulation with recruitment *CV* = 1.0 and autocorrelation in recruitment *ρ* = 0.5, all combinations of harvest rates yield fishery closures more than 10% of the time (Fig. 5b). Thus, even in the absence of fishing, recruitment variation will cause populations to, occasionally, decline to low levels and cause a fishery closure (e.g. Fig. 1a, 1d).

**Figure 6:**
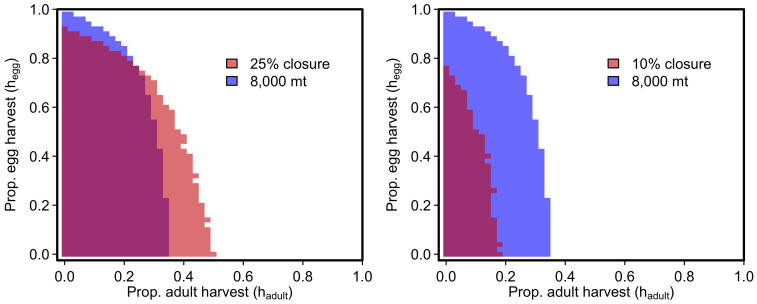
Risk plot comparing the probability of fisheries closure and *B_ecosystem_* for all combinations of egg and adult harvest. Shaded areas indicate harvest levels that satisfy the risk of fishery closure (pink; <25% probability in closure in left panel, <10% probability of closure in right panel) or average herring biomass is more than *B_ecosystem_* = 8,000 mt (blue in both panels). Harvest rates satisfying both criteria are shown in purple. Both plots show results for recruitment variability of *CV* = 0.8 and *ρ* = 0.5.

In addition to the backdrop of natural environmental variation, herring fisheries, like many targeting forage fishes, are increasingly asked to account for ecosystem needs in determining appropriate harvest rates. Our model predicts that many combinations of egg- and adult-harvest rates can allow the herring fishery to remain open while at the same time maintaining average herring biomass above *B_ecosystem_*, an ecosystem threshold intended to leave enough herring biomass in the water to satisfy the needs of herring predators (purple shaded area in Fig. 6). However, maintaining herring biomass levels above *B_ecosystem_* imposes a greater constraint on the adult fishery than a hypothetical management target that seeks to avoid fishery closures more than 25% of the time (pink areas in Fig. 6a). In contrast, the 25% fishery closure rule would exclude very high levels of *h_egg_* but a goal of maintaining herring biomass levels above *B_ecosystem_* would not (blue areas in Fig. 6a). Interestingly, a hypothetical management target that avoids fishery closures more than 10% of the time placed a stronger constraint on both adult and egg harvest rates than did *B_ecosystem_* (the lack of pink in Fig. 6b indicates the 10% closure rule always limits harvest rates). Therefore, harvest rate combinations that avoid fishery closures more than 10% of the time would always be consistent with those that satisfy this choice of *B_ecosystem_* (Fig. 6b).

## Discussion

Ecologists and fisheries scientists have long recognized that changes in the densities of strongly interacting species have disproportionately large influences on the communities and ecosystems of which they are a part^28^,^29^. In the marine realm, it is increasingly apparent that forage fish can play this key role and that fisheries targeting them therefore require special consideration^22^,^27^. But awareness of the big impact these little fishes can have in general provides little guidance on how different types of human activities and natural environmental variation combine to modify their dynamics in more specific and management-relevant contexts. Developing such an understanding is a necessary step toward providing conservationists and fisheries managers with the counsel they need to make informed decisions about the sustainability and impacts of their actions.

This study adds to a growing body of work showing that stage-specific exploitation can have disproportionately strong or weak effects on population dynamics, depending on the point in the life history at which it occurs^8^,^30^,^31^. It also underscores the potential for conflict among user groups that target different life stages. Specifically, our model predicts that egg and adult fishing have nonequivalent effects on herring population dynamics and fisheries yields. Harvest of adult herring reduced mean spawning biomass, and increased variability in herring spawning biomass, much more so than harvesting eggs (Fig. 2). As a consequence, increasing harvest of adult herring caused precipitous declines in egg catch, whereas increasing harvest of herring eggs produced much slower declines in adult catch (Fig. 3a, 3b). This asymmetry arises in part because of the order in which harvest occurs - reproductively mature adult fish must be caught before they spawn - and in part because of the density-dependent nature of recruitment. Relatively low numbers of eggs can still produce substantial number of recruits two years later.

However, decisions about how much harvest is too much harvest on any one life stage are complicated by many factors, not the least of which are socio-economic and political consequences generated by biological constraints. For the parameter values used in our case study, we see that herring populations are much more sensitive to adult harvest. High levels of adult harvest are more likely to lead to an increase in the frequency and duration of fishery closures than high levels of egg harvest. Qualitatively, we suspect that this is a general result for most harvested fishes and other harvested, long-lived species (ungulates, trees; see e.g.^32^,^33^). However, we note that the effect of different types of harvest of a given species or population within a species will depend strongly on the biological details of recruitment, growth, and mortality.

The crux of EBFM is trade-offs—trade-offs among fisheries sectors or between fisheries and other objectives, such as conservation. Thus, a general contribution of this work is providing methods for visualizing and presenting trade-offs between alternative fisheries harvest regimes and other ecosystem goals. Our work provides a forum for explicitly discussing metrics of biological, social, and economic risk in the management of marine systems. Embracing and explicitly acknowledging the complexity of ocean management provides avenues for considering how alternative management strategies affect different stakeholder groups. For example, social disruption caused by a particular management action (or lack thereof) or a natural catastrophe may have non-equivalent effects on different components of a fishery (e.g.^34^,^35^,^36^,).

Operationalizing ecosystem-based management requires that we not only confront allocation issues among herring fisheries sectors, but fisheries managers must also tackle calls for an allocation of herring to the ecosystem. Perhaps what is most striking about our model predictions, however, is that ecosystem thresholds proposed to ensure the persistence of herring predators do not necessarily pose more stringent constraints on fisheries than these fisheries pose on themselves via hypothetical (but realistic) management targets. Thus, an important lesson from these analyses is that the safe operating space^37^ for fishermen and conservationists can be similar, and can aid in facilitating management actions to ensure fisheries and ecosystem needs are met in the long-term (cf.^38^). We note, however, that the choice of *B_ecosystem_* and the acceptable frequency of fishery closures are political choices that can be informed but not dictated by scientific information. Furthermore, there are biological assumptions that influence the nature of trade-offs among fisheries and conservation goals; the values we provide here are intended to be illustrative, not definitive.

Our work is meant to strategically explore the consequences of different harvest regimes in the face of varying environmental conditions. The uncertainties and simplifications inherent in our modeling framework make it inappropriate for tactical use. In particular, we based our simulations on reasonable values of stochastic recruitment that bracket current uncertainty about recruitment variability. However, as Fig. 5 illustrates, small differences in assumed recruitment variability can radically affect the fisheries risk profiles (also see Figs. A1–A4). Beyond recruitment, our model does not incorporate measurement uncertainty– fish biomass and catch are assumed to be precisely known. While the simulation and visualization methods used in this paper are robust to the inclusion of measurement error, the process of measuring fish biomass and catch adds a further layer of uncertainty to the management of fisheries^39^.

Our simulations show how natural environmental variation can cause herring fisheries closures in the absence of harvest (e.g. Fig. 1a), contrasting with^40^ who use archeological data to assert hyperstability in herring populations. Our results are consistent with basic population dynamic principles and show how environmental variation, in the absence of human influence, affects population persistence^41^. Thus our model agrees with other authors in showing that it is impossible to accurately generate harvest guidelines without accounting for changes in the environment in which a stock occurs^42^, and reinforces the challenge faced by fisheries managers to make good decisions under uncertainty about current environmental conditions.

At its core, ecosystem management must confront conflict. In this case, we explore the conflicts inherent in fisheries on different life history stages, and conflicts intrinsic to forage fishes that are both important fishery targets and ecosystem components. Our work highlights that safe operating spaces exist that will allow the maintenance of stock biomass at levels that facilitate the persistence of forage fish predators and forage fish fisheries. Importantly, however, our work also emphasizes the fact that many trade-offs are non-linear, as is illustrated in the asymmetry in the trade-off between the harvest of herring eggs and adults. We contend that an analysis of risk- whether of a fishery closure or to another ecosystem component (e.g. mammals or birds)- provides a transparent and straightforward decision support tool to address such non-linearities. Thus, this tool can highlight opportunities for collaboration and cooperation among conservationists and those in the fisheries sectors. It also offers the chance to avoid unintended consequences on non-target life stages and species. As this case study of Pacific herring illustrates, the science to inform ecosystem-based fisheries management is available, but it is up to decision makers to use it in a way that strikes a balance between the competing needs of multiple stakeholders and species.

## Methods

We simulated Pacific herring populations under different levels of adult and egg harvest, *h_adult_* and *h_egg_*, respectively (*h*. represents the proportion of each stage harvested annually and bounded between 0 and 1) using a stochastic age-structured model. We investigate the consequences of the two types of harvest on the abundance of herring, the catch of adult herring and eggs, and the frequency with which the population declined below the biomass required to open the fishery, *B_lim_*. Our simulations are intended to reflect the potential population trajectories over the near term. Therefore we simulate populations that start at low biomass (reflecting current conditions of some herring stocks) over the next 40 years.

A major uncertainty and determinant of herring populations is the variability of recruitment^21^,^22^. To account for our uncertainty about recruitment, we simulated all harvest strategies across nine distinct recruitment scenarios that reflect biologically plausible ranges of recruitment variability and temporal autocorrelation. Figure 1 shows examples of simulated biomass time-series for four scenarios: two comparing populations in the absence and presence of harvest (Fig. 1a, 1b, respectively) and two comparing unfished populations under distinct recruitment regimes (Fig. 1c, 1d).

### Population model for herring

To investigate the consequences of alternative fishing strategies for population dynamics and harvest, we constructed an age-structured model of herring populations. Following convention for Pacific herring, male and female herring are assumed to have identical vital rates^43^,^44^,^45^ and we only model the dynamics of female fish.

Let *X_a,t_* indicate the number of female herring of age *a* in year *t*. Because the fishery on adult herring occurs over a very short period immediately before the herring spawn, we model survival from immediately after spawning to immediately before the herring fishery. The number of adult herring of age *a* in year *t* before spawning, *X_pre,a,t_*, is a function of the number of herring that survived to spawn in the previous year, and the density-independent natural mortality rate, *M*. 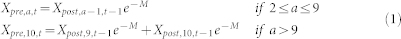
where *post* designates the population immediately after harvest and the second line represents a plus group that includes all individuals age 10 and older.

Only a subset of the herring population returns to spawn grounds each year so we distinguish between the total number of herring in the population, *X*, and those that are mature and return to the spawning grounds to spawn, *Y*. As herring are harvested for their roe, only mature individuals present on the spawning grounds are subject to harvest. The proportion of age *a* individuals that mature and return to spawn is *p_a_*. Thus the number of mature individuals vulnerable to the fishery is 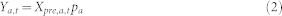
and the biomass present on the spawning grounds is 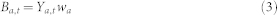
where, *w_a_* is the weight-at-age. Note that both the maturity-at-age and weight-at-age are constant across the simulation.

There are two distinct fisheries for spawning herring. The first targets mature, spawning adults and captures a proportion of the spawning biomass, *h_adult_*. The second fishery targets herring eggs after they have been deposited on shallow coastal vegetation including macroalgae (e.g. *Laminaria spp.*) or eelgrass (*Zostera marina*). The fraction of eggs harvested each year is *h_egg_*. Both *h_adult_* and *h_egg_* are bounded between 0 and 1. Harvest control rules dictate that the fishery for herring closes when spawning biomass falls below a threshold biomass, *B_lim_* (defined below). Since herring are harvested before they spawn, the catch, *C*, and biomass of fish after the fishery is 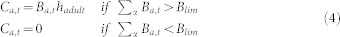
And so the biomass surviving to spawn is 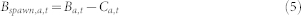
We assume that spawning herring are equally vulnerable to harvest (fishing is not age selective) as is consistent with purse seine fisheries for herring. The number of eggs produced by age *a* spawning adults, is related to the weight-specific fecundity of each age (*f_a_*, eggs per gram of herring), 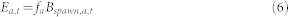
The second fishery occurs directly on herring eggs. We assume the same harvest control rules as above; the fishery closes if adult spawning biomass before harvest is less than *B_lim_*. Then, 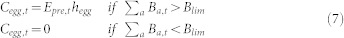
and the eggs remaining after harvest 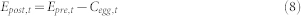
Finally, the recruitment of age two individuals to the population follows a Beverton-Holt recruitment relationship. 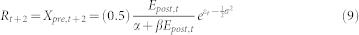
Here, *α* and *β* are parameters of the stock-recruitment relationship and *ε* ~ *N*(0,*σ*^2^) controls recruitment deviations from the deterministic stock-recruitment relationship. We describe recruitment stochasticity in the next section. The 0.5 is included because half of the population is male and we only track female fish. By writing eqn. 9 in terms of the post harvest egg abundance, we assumed that density-dependence in recruitment occurs after egg harvest.

While this model is a general representation of any herring population, we use parameter values that approximate the herring population in Haida Gwaii, British Columbia, to illustrate of herring population dynamics and fisheries. Table 1 lists the parameter values used in all simulations and their literature sources.

### Herring model simulations

In this section, we describe stochastic simulations designed to investigate the consequences of alternative harvesting regimes for herring populations and fisheries. We are interested in the interaction between the fishery on adult herring and the harvest of herring eggs in the presence of environmental variation. We first discuss the population reference points before outlining harvesting scenarios and describing the nine environmental scenarios.

Defining fisheries reference points is difficult and complex. We used a simple approach to establish a reference point for all subsequent simulations. We defined herring biomass in the absence of fishing (unfished biomass, *B_0_*) using the deterministic version of the age-structured model (i.e. 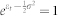). We simulated the age-structured model with *h_egg_* = *h_adult_* = 0 until it converged on an equilibrium biomass (using parameter values in Table 1; *B_0_* ≈ 24,000 mt of female herring). Following the harvest control rules used in the British Columbia herring fisheries^46^, we defined a biomass limit at which the fishery closed as 25% of unfished biomass (*B_lim_ = 0.25B_0_*).

To understand the consequences of different harvest strategies, we simulated populations assuming different combinations of *h_adult_* and *h_egg_* over a 40 year time span. We simulated populations with a fixed *h_adult_* and fixed *h_egg_*; all combinations of *h_adult_* and *h_egg_* between 0.0 and 0.98 in increments of 0.02 were investigated. We started the population with a standard biomass and age-structure in year 0 (Table 1) and allowed fishing to begin in year 1. For a given environmental variability scenario, we repeated each combination of *h_adult_* and *h_egg_* 1000 times. Our simulations assume perfect knowledge about the biomass of spawning adults and the number of spawned eggs such that the exact proportion of adult biomass and eggs were harvested. This is an unrealistic assumption in practice –natural populations will always have uncertainty in the size of the population and in the amount of herring captured by the fishery. However, such simplifying simulations are appropriate for understanding the general consequences of different levels of adult and egg harvest.

Herring populations, like many short-lived forage fishes, are dominated by variation in recruitment^21^,^22^. While the mechanistic drivers of recruitment variability remain largely unknown, we approximate the variability observed in natural populations by making recruitment a stochastic process. We incorporated recruitment variation using three levels of stochastic variation. We express the variability of recruitment in terms of the coefficient of variation (*CV*), a non-dimensional measure of variability comparable across stocks and species (for random variable *Z*, 
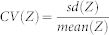
). We simulated populations under three levels of recruitment variability 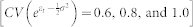. Available data indicate that Pacific herring have recruitment *CV* of approximately 0.8^46^,^47^; therefore, our simulations span a plausible range of recruitment variability.

In addition to variability, environmental conditions are often autocorrelated causing adjacent recruitment years to be similar to one another. Such autocorrelation leads to the occurrence of environmental regimes and extended periods of favorable or unfavorable recruitment. For example, the Pacific Decadal Oscillation (PDO^48^,^49^) is often invoked as a large-scale driver of Pacific oceanic conditions. The PDO has a lag-one autocorrelation (AR(1)) of approximately 0.5, indicating large scale temporal autocorrelation in the ocean conditions of the North Pacific ocean. While no direct estimates of autocorrelation in herring recruitment are available, we investigated three scenarios, AR(1) = *ρ* = 0.0, 0.5, and 0.7. In terms of the model described above, recruitment deviations are then *ε_t_* ~ *N*(*ρε_t_*_−1_,*σ*^2^).

We simulated 1000 independent populations for 40 years using each combination of egg and adult harvest (2500 combinations in total) and all nine pairs of recruitment variation (three CV levels and three levels of autocorrelation). We refer to each set of harvest rates and recruitment variation as a scenario. For each simulation, we recorded the mean and standard deviation in spawning biomass, and catch of eggs and adults. We report the mean of these 4 response variables across the simulations in each scenario. In addition, we kept track of the frequency with which the population declined below the fishery closure limit (*B_lim_*).

Several recent studies have emphasized the reliance of marine predators on forage fish prey, suggesting that one-third to three-quarters of forage fish biomass are required to prevent predator population declines^20^,^27^. Therefore, we also tracked whether each set of simulated harvest rates would allow for the maintenance of an average herring spawning stock biomass above an ecosystem threshold, *B_ecosystem_* (“one-third for the birds” or 8,000 mt).

## Author Contributions

A.O.S., J.F.S., A.C.S. and P.S.L. contributed to the design and development of the manuscript. A.O.S. and J.F.S. wrote the main manuscript text. A.O.S., J.F.S., A.C.S. and P.S.L. reviewed and edited the manuscript and figures.

## Supplementary Material

Supplementary Information Supplementary Information

## Figures and Tables

**Table 1:** Parameters used in stochastic simulations of herring populations

Age	Maturity* (*p_a_*)	Weight† (*w_a_*, grams)	Specific Fecundity‡ (*f_a_*, eggs gram^−1^)	Year 0 Biomass (*B_a,0_*)	Year 0 Spawning Biomass (*B_spawn,a,0_*)
2	0.25	51.76	134.98	3916	979
3	0.85	70.51	153.33	2004	1703
4	0.92	87.56	163.20	1025	943
5	0.95	104.39	169.78	525	498
6	0.967	116.47	173.33	268	260
7	0.98	126.41	175.74	137	135
8	0.99	138.34	178.18	70	70
9	0.995	144.33	179.25	36	36
10+	0.999	152.28	180.54	18	18
Natural Mortality†(*M*)				0.67	
Unfished Biomass (*B_0_*)				23,914	
Harvest Closure (*B_lim_*)				5,979	
Initial Biomass 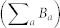				8,000	
Initial Spawning Biomass 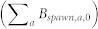				4,641	
**Recruitment**†					
α				0.4187	
β				0.001883	

*derived from^50^.

^†^derived from^46^.

^‡^derived from weight-fecundity relationships presented in^51^ (Table 2, pg. 20).
