# Supplementary material for: Assessing trade-offs to inform ecosystem-based fisheries management of forage fish
Source: Sci Rep. 2014 Nov 19;4:7110. doi: 10.1038/srep07110 (PMC4236757; doi:10.1038/srep07110)
Supplement: Supplementary Information [file srep07110-s1.pdf]

Supplement to:

# Assessing trade-offs to inform ecosystem-based fisheries management of forage fish

Andrew Olaf Shelton*

Jameal F. Samhouri

Adrian C. Stier

Philip S. Levin

Conservation Biology Division, Northwest Fisheries Science Center, National Marine Fisheries Service, National Oceanic & Atmospheric Administration, Seattle, WA

* Corresponding author: ole.shelton@noaa.gov

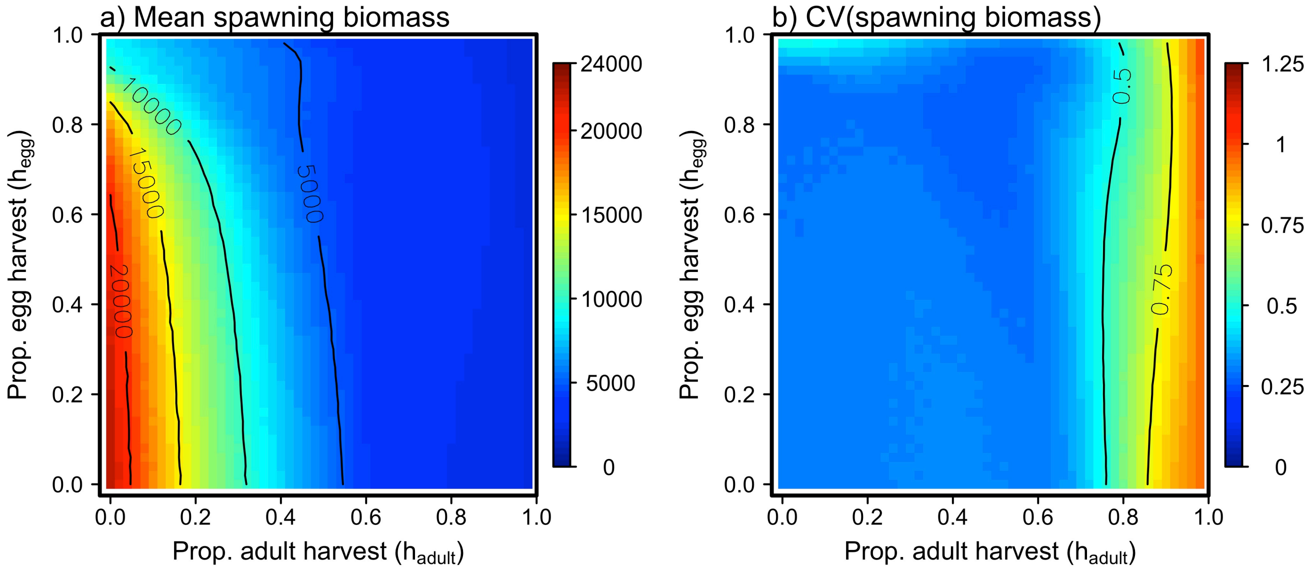


Figure A1: Consequences of adult and egg harvest strategies on mean spawning biomass (*a*) and the coefficient of variation in spawning biomass (*b*). Both panels show results for CV = 0.6, and $\rho = 0.0$.

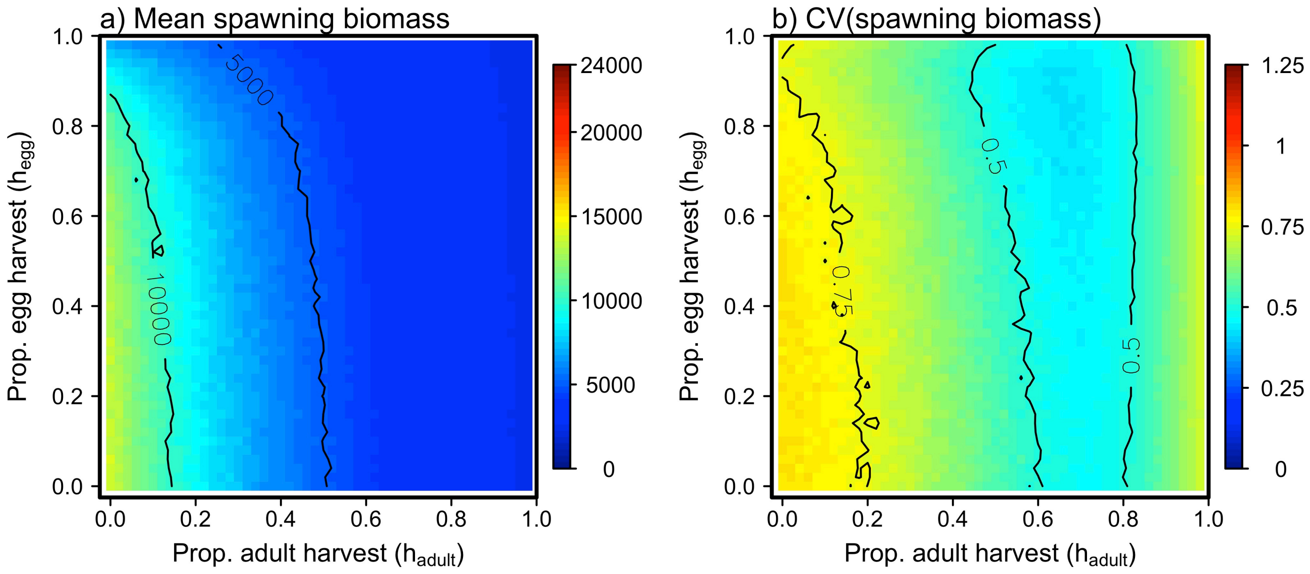


Figure A2: Consequences of adult and egg harvest strategies on mean spawning biomass (*a*) and the coefficient of variation in spawning biomass (*b*). Both panels show results for CV = 1.0, and $\rho = 0.7$.

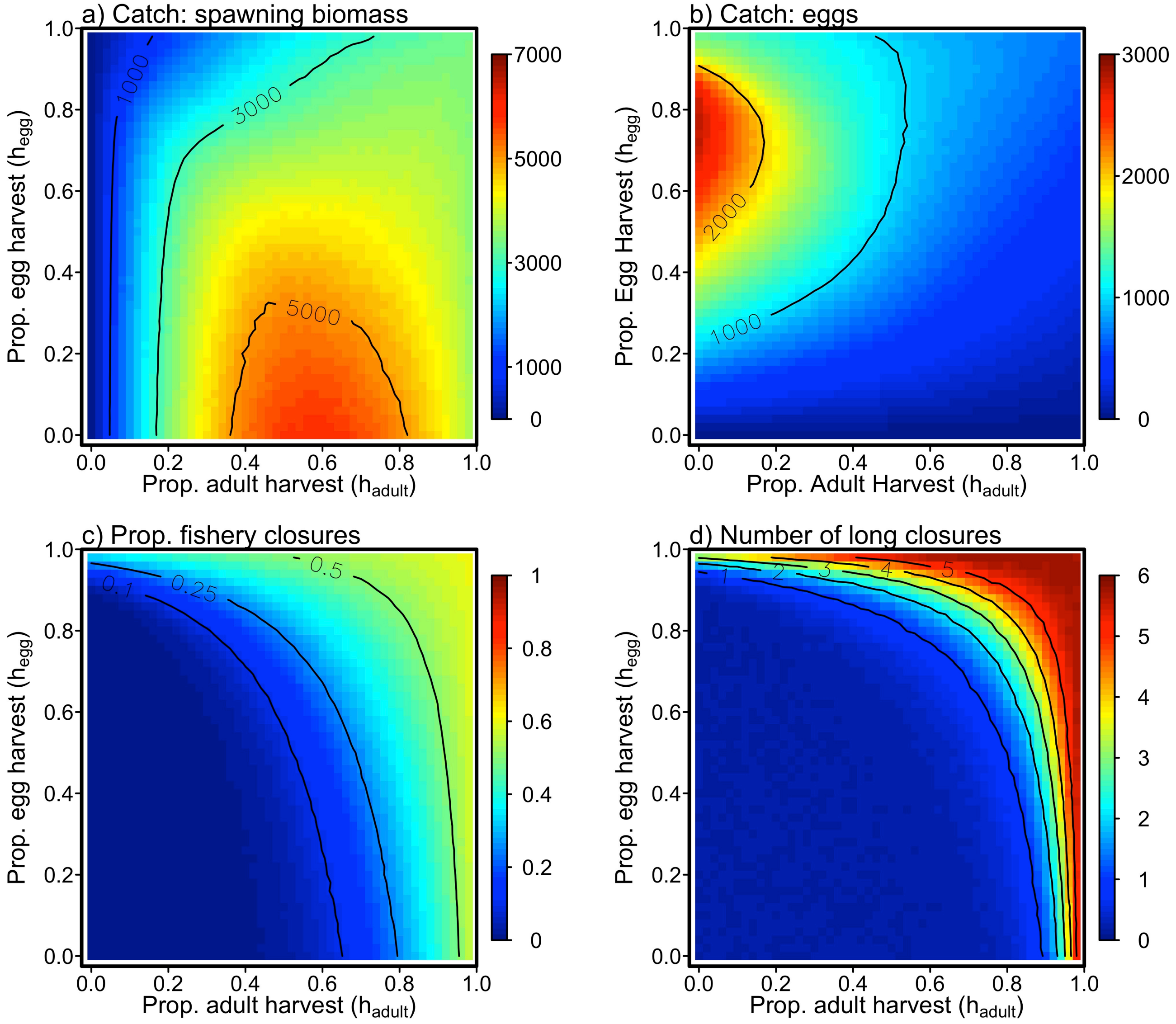


Figure A3: Consequences of adult and egg harvest strategies on the herring fisheries. *a*) Mean catch of spawning biomass (mt), *b*) mean catch of eggs (trillions), *c*) the proportion of years that the fishery is closed, and *d*) mean number of long closures (>3 consecutive years closed) in the 40 year simulation. CV= 0.6, $\rho = 0$.

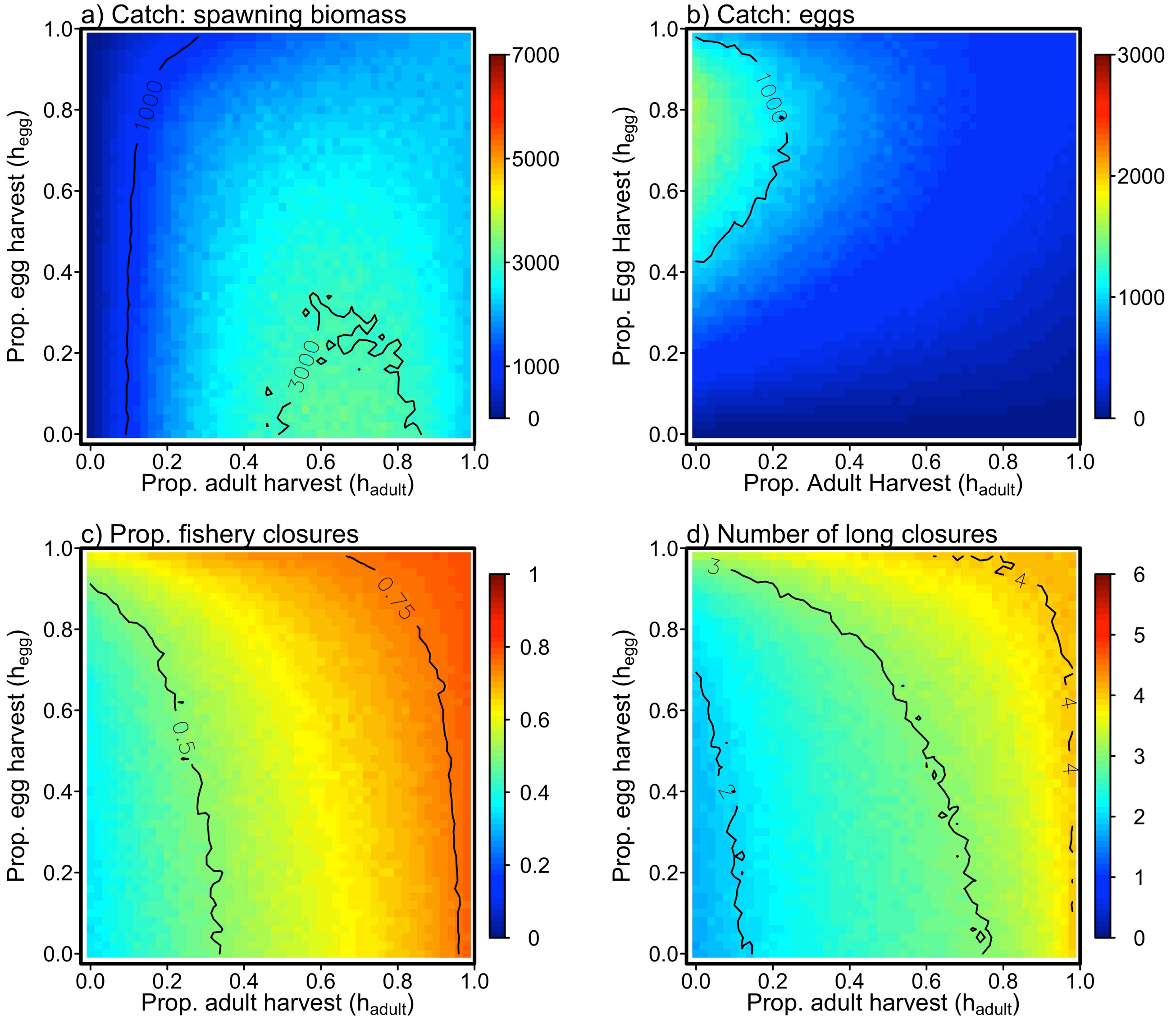


Figure A4: Consequences of adult and egg harvest strategies on the herring fisheries. *a*) Mean catch of spawning biomass (mt), *b*) mean catch of eggs (trillions), *c*) the proportion of years that the fishery is closed, and *d*) mean number of long closures (>3 consecutive years closed) in the 40 year simulation. $CV = 1.0$, $\rho = 0.7$

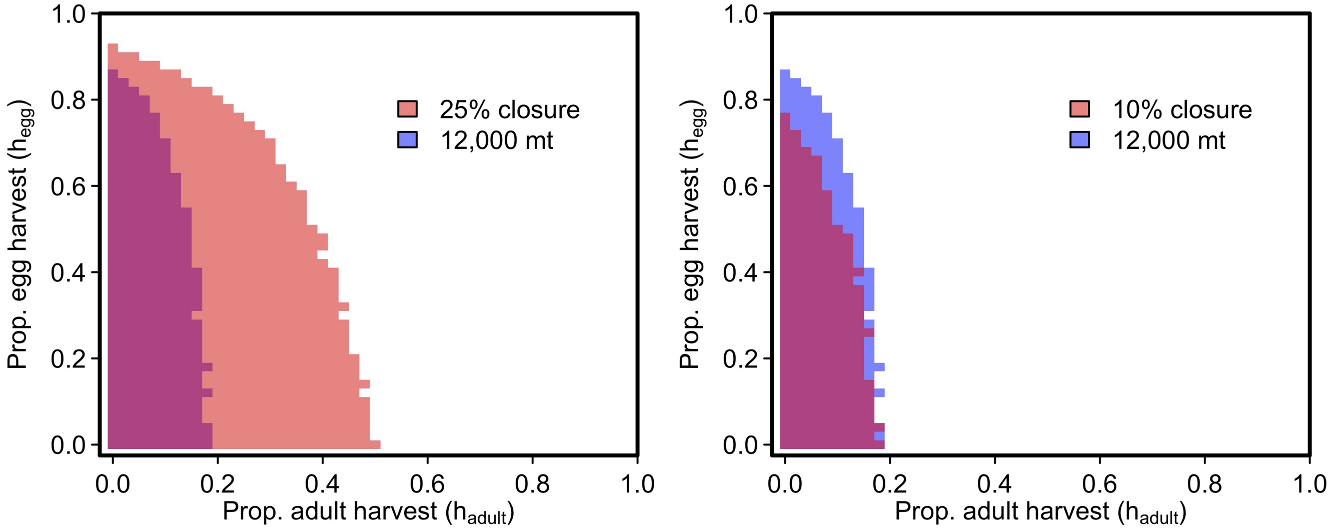


Fig A5. Risk plot comparing the probability of fisheries closure and $B_{ecosystem}$ for all combinations of egg and adult harvest. Shaded areas indicate harvest levels that satisfy the risk of fishery closure (red; <25% probability in closure in left panel, <10% probability of closure in right panel) or average herring biomass is more than $B_{ecosystem}$ = 12,000 mt (blue in both panels). Harvest rates satisfying both criteria are shown in purple. Both plots show results for recruitment variability of $CV$=0.8 and $\rho$=0.5.
